# Distinct phosphorylation sites on the ghrelin receptor, GHSR1a, establish a code that determines the functions of ß-arrestins

**DOI:** 10.1038/srep22495

**Published:** 2016-03-03

**Authors:** Monica Bouzo-Lorenzo, Icía Santo-Zas, Maria Lodeiro, Rubén Nogueiras, Felipe F. Casanueva, Marian Castro, Yolanda Pazos, Andrew B Tobin, Adrian J. Butcher, Jesús P. Camiña

**Affiliations:** 1 Área de Endocrinología Molecular y Celular, Instituto de Investigación Sanitaria de Santiago (IDIS), Complejo Hospitalario Universitario de Santiago (CHUS), Servicio Gallego de Salud (SERGAS), Santiago de Compostela, Spain; 2 CIBER Fisiopatología de la Obesidad y Nutrición, Spain; 3 Departamento de Fisiología, Universidad de Santiago de Compostela (USC), Santiago de Compostela, Spain; 4 Departamento de Medicina, USC, Santiago de Compostela, Spain; 5 Center for Research in Molecular Medicine and Chronic Diseases (CIMUS), USC, Santiago de Compostela, Spain; 6 Medical Research Council Toxicology Unit, University of Leicester, Hodgkin Building, Lancaster Road, Leicester, LE1 9HN, United Kingdom

## Abstract

The growth hormone secretagogue receptor, GHSR1a, mediates the biological activities of ghrelin, which includes the secretion of growth hormone, as well as the stimulation of appetite, food intake and maintenance of energy homeostasis. Mapping phosphorylation sites on GHSR1a and knowledge of how these sites control specific functional consequences unlocks new strategies for the development of therapeutic agents targeting individual functions. Herein, we have identified the phosphorylation of different sets of sites within GHSR1a which engender distinct functionality of ß-arrestins. More specifically, the Ser^362^, Ser^363^ and Thr^366^ residues at the carboxyl-terminal tail were primarily responsible for ß-arrestin 1 and 2 binding, internalization and ß-arrestin-mediated proliferation and adipogenesis. The Thr^350^ and Ser^349^ are not necessary for ß-arrestin recruitment, but are involved in the stabilization of the GHSR1a-ß-arrestin complex in a manner that determines the ultimate cellular consequences of ß-arrestin signaling. We further demonstrated that the mitogenic and adipogenic effect of ghrelin were mainly dependent on the ß-arrestin bound to the phosphorylated GHSR1a. In contrast, the ghrelin function on GH secretion was entirely mediated by G protein signaling. Our data is consistent with the hypothesis that the phosphorylation pattern on the C terminus of GHSR1a determines the signaling and physiological output.

The growth hormone secretagogue receptor type 1a (GHSR1a), a receptor belonging to the diverse group of seven transmembrane receptors, critically regulates the central and peripheral actions of ghrelin as a growth hormone secretagogue, an orexigenic peptide, and a long-term regulator of energy homeostasis^1^. Ghrelin is a 28-amino acid residue peptide with a post-translational octanoyl modification on Ser3, which was first discovered in rat and human stomach tissues^2^. This hormone is mainly synthesized in the stomach, but substantially lower amounts have been detected in other tissues^3^,^4^. GHSR1a is expressed in several brain areas, including the anterior pituitary gland where it stimulates the release of growth hormone (GH)^2^,^5^ as well as the hippocampus and hypothalamic arcuate nucleus where it regulates feeding behavior^6^,^7^, and the *substantia nigra pars compacta*, ventral tegmental area and raphe nuclei where GHSR1a controls the mesolimbic dopaminergic reward circuit^1^,^8^. At peripheral level GHSR1a is expressed in pancreatic islets, adrenal gland, thyroid, myocardium, and adipose tissue. Numerous peripheral actions of ghrelin have been described which include regulation of glucose metabolism, lipogenesis, suppression of brown fat thermogenesis and improvement of cardiovascular functions such as vasodilatation and cardiac contractility^1^.

GHSR1a classically exerts intracellular effects through G-protein activation, mainly via G_q/11_ and G_i/o_^9^,^10^. However, recent evidence has demonstrated that ß-arrestins act as molecular mediators of G-protein independent signaling by acting to scaffold a variety of signaling proteins^11^,^12^,^13^,^14^. Initially, described as contributing to desensitization of GHSR1a through the regulation of the endocytosis^15^ ß-arrestins are now known contribute to GHSR1a signal transduction via ERK1/2-mitogen-activated protein kinase^11^ and Akt/protein kinase B^12^,^13^,^14^ signaling pathways. The signaling mechanisms that underlie the activation of the ERK response by GHSR1a are complex and the result of both classical G protein signaling and ß-arrestin–dependent processes^12^. The G protein component of this response involves G_i/o_-dependent signaling upstream of phosphatidylinositol 3-kinase (PI3K), protein kinase Cε, and the non-receptor tyrosine kinase cSrc as well as G_q/11_-dependent signaling involving the activation of protein kinase Cα/ß and cSrc. These G protein-dependent mechanisms operate in concert with GHSR1a mediated ß-arrestin signaling that lies up-stream of cSrc, Raf-1, and ERK1/2.

In addition, GHSR1a activates Akt signaling through a complex interplay of distinct signaling mechanisms: an early G_i/o_ protein–dependent pathway and a late pathway mediated by ß-arrestins. The early G_i/o_ protein–dependent pathway involves PI3K activation that leads to the membrane recruitment and activation of Akt. The later phase of Akt activation is dependent on ß-arrestins 1 and 2 and involves the recruitment of cSrc and Akt into a complex. This ß-arrestin-scaffolded complex leads to full activation of Akt through PDK1 and mTORC2, which are not physically associated to the complex. Whether GHSR1a activates Akt through the G protein pathway or the ß-arrestin-dependent pathway is determined by cSrc, which functions as a switch. This switch operates by cSrc phosphorylating the C-terminus of SHP-1 (Tyr536), which results in an inhibitory effect on G_i/o_ protein activation of Akt by inhibiting the activity of PI3K^12^,^13^.

Recently we have established the importance of ß-arrestin-mediated signaling in functional ghrelin/GHSR1a responses. In particular, adipogenic functions of the ghrelin/GHSR1a system have been linked with ß-arrestin signaling. In these studies ß-arrestin depletion during ghrelin-induced adipogenesis reduced C/EBPß, C/EBPδ, C/EBPα and PPARγ levels, leading to a significant reduction of lipid accumulation and to the impairment of terminal differentiation^14^.

Thus, it is becoming increasingly evident that ß-arrestins, originally discovered as mere adaptor proteins for the GHSR1a endocytosis, have much broader signaling and physiological roles. What is not clear however are the factors that regulate GHSR1a coupling to ß-arrestin-dependent signaling and the processes that regulate the relative contribution of G-protein versus ß-arrestin-dependent signaling. It is now well established that GPCR phosphorylation plays a crucial role in the recruitment and activation of ß-arrestin-dependent signaling. Recent studies have gone further and suggested that the pattern of phosphorylation on a GPCR constitutes a barcode that determines, at least in part, the signaling outcomes^16^,^17^,^18^,^19^,^20^. The possibility that GHSR1a regulates ß-arrestin-dependent signaling through a phosphorylation barcode, has, however, not been investigated. Here we determine the phosphorylation sites on GHSR1a and show that these are arranged into two distinct clusters on the C terminus of the receptor. By analysis of the impact that GHSR1a phosphorylation has on various signaling and cellular responses we provide evidence of a phosphorylation barcode that instructs the recruitment of ß-arrestin and determines GHSR1a functionality.

## Results

### Identification of phosphorylation sites in GHSR1a by mass spectrometry

**Figure 1:**
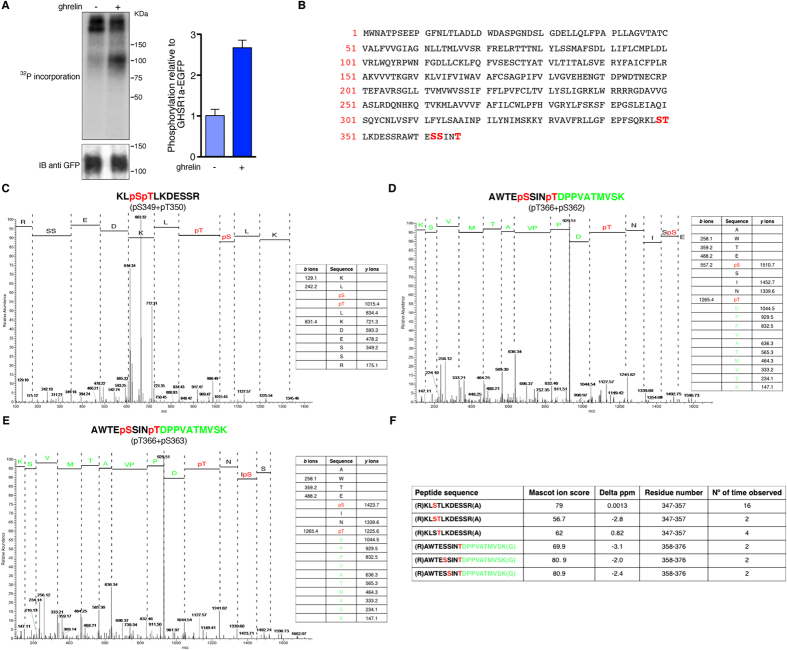
Mass spectrometry identifies five distinct sites of phosphorylation in the GHSR1a. HEK 293 cells transiently expressing C-terminally EGFP-tagged GHSR1a were either labeled with ^32^P (**A**) or used to immunoprecipitate and then digest the receptor for analysis using mass spectrometry (**C**–**E**). In the ^32^P labeling studies, cells were treated with the agonist ghrelin (100 nM) or vehicle for 5 min prior to sample preparation. (**A**) *Left panel*, autoradiograph and loading control (EGFP immunoblot) is shown. *Right panel*, levels of ^32^P were quantified by densitometry, normalized to GHSR1a-EGFP, and expressed as fold increase relative to the control cells. Immunoblot is representative of five independent experiments. The data are expressed as the mean ± SE (*p < 0.05). (**B**) Amino acid sequence of the GHSR1a indicating in red the amino acids identified as being phosphorylated. (**C**–**E**) representative mass spectra and associated fragmentation tables are shown for the five phosphorylated amino acid residues, three serine (Ser^349^, Ser^362^ and S^363^) and 2 threonine (Thr^350^ and Thr^366^), which were identified. Green text denotes amino acid residues identified from eGFP. (**F**) Summary of the overall data set.

**Figure 2:**
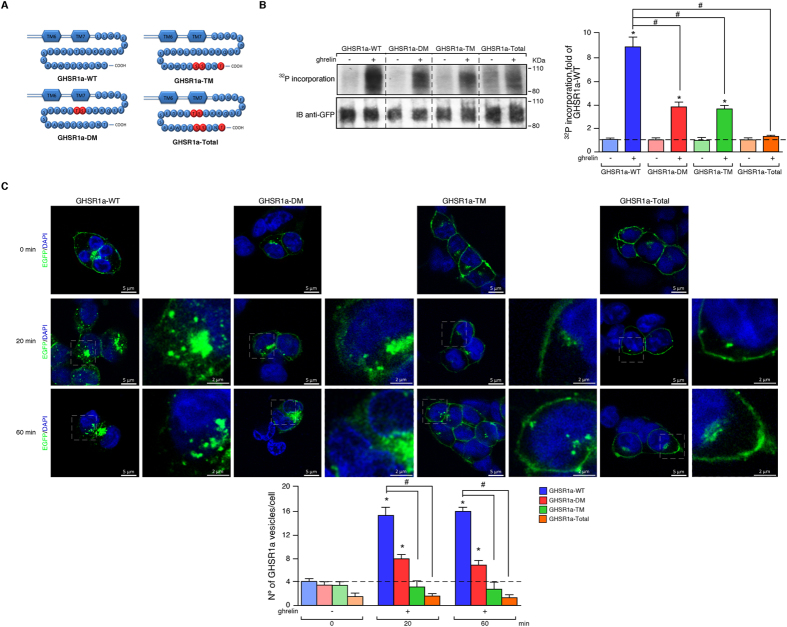
Mutational analysis of GHSR1a on ghrelin-induced phosphorylation and endocytosis. (**A**) Schematic representation of the C-terminal portion of GHSR1a showing the Ser and Thr residues that were found to be phosphorylated and that were subsequently mutated to alanine to generate GHSR1a-DM, GHSR1a-TM and GHSR1a-Total mutants. The mutated residues are shown in red. (**B**) *Left panel*, ^32^P labeling studies were performed using HEK 293 cells transiently expressing either the EGFP-tagged GHSR1a-WT or the mutants. *Right panel,* levels of ^32^P were quantified by densitometry, normalized to GHSR1a-EGFP, and expressed as fold increase relative to the control cells expressing the GHSR1a-WT. Immunoblots are representative of five independent experiments. The data are expressed as the mean ± SEM (^*,#^*p* < 0.05). (**C**) Analysis of GHSR1a endocytosis by confocal microscopy. EGFP-tagged GHSR1a-WT or C-terminal tail Ala mutants were transiently expressed in HEK 293 cells and stimulated with ghrelin (100 nM) for stated times at 37 °C. In absence of ligand, the fluorescent labeling appeared at the cell surface for the GHSR1a-WT and mutants. After a 20 and 60 minutes stimulation with ghrelin, the extent of receptor endocytosis is substantially reduced in the GHSR1a-DM, GHSR1a-TM and GHSR1a-Total mutants compared to the GHSR1a-WT (lower panel). Confocal images are representative of three independent experiments. The data are expressed as the mean ± SEM (^*,#^*p* < 0.05).

Following stimulation with ghrelin (100 nM, 5 min), phosphorylation of GHSR1a was enhanced as monitored by increased incorporation of ^32^P into a protein with an apparent molecular mass ∼100 KDa (~2.8 ± 0.2-fold; Fig. 1A). To identify the precise phosphorylation sites, a mass spectrometry-based proteomics study of tryptic peptides generated from the isolated GHSR1a was conducted (Fig. 1B–E). These studies revealed 3 serine (Ser^349^, Ser^362^ and Ser^363^ and 2 threonine (Thr^350^ and Thr^366^) phospho-acceptor sites at the C-terminal tail. The tryptic peptides generated by digestion of GHSR1a included peptides originating from the third intracellular loop, however, there was no indication that any of the peptides from this region were phosphorylated. HEK cells stably expressing GHSR1a were generated in which these phosphorylation sites were mutated to Ala residues. In a double mutant of GHSR1a, designated GHSR1a-DM, in which Thr^350^ and Ser^349^ were mutated to Ala, phosphorylation in response to ghrelin was reduced by 57±3% (Fig. 2B). Triple mutation of GHSR1a, designated GHSR1a-TM, in which Ser^362^, Ser^363^and Thr^366^ were mutated to Ala, phosphorylation was reduced by 58±2% (Fig. 2B). A further mutant was generated in which all of the residues identified by mass spectrometry to be phosphorylated were substituted by Ala (Ser^349^, Ser^362^, Ser^363^, Thr^350^ and Thr^366^), and was designated GHSR1a-Total. The phosphorylation status of GHSR1a-Total was significantly less than that of either GHSR1a-DM or GHSR1a-TM indicating that the sites of ghrelin-regulated phosphorylation in the GHSR1a were mainly Ser^349^, Ser^362^, Ser^363^ Thr^350^ and Thr^366^.

### Phosphorylation of the C-terminal tail of the GHSR1a regulates receptor endocytosis and ß-arrestin 1 and 2 recruitment

**Figure 3:**
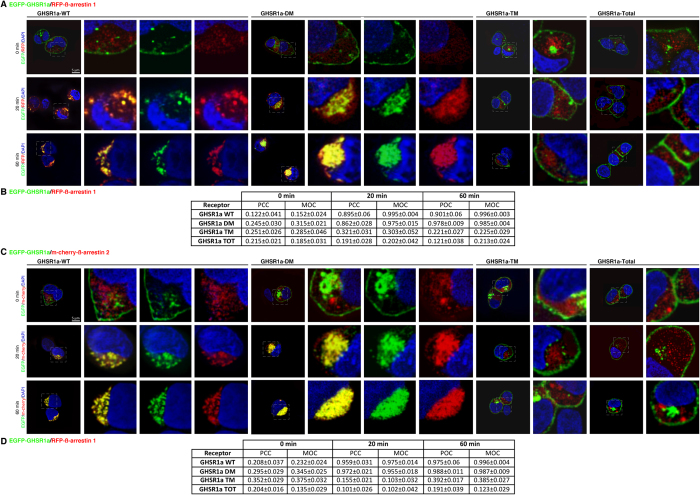
The phospho-acceptor residues in C-terminal tail of GHSR1a-WT govern both binding and trafficking patterns of ß-arrestins. (**A**) Trafficking of the RFP-tagged ß-arrestin 1 with the EGFP-tagged GHSR1a-WT or the mutants GHSR1a DM, GHSR1a -TM or GHSR1a TOTAL expressed in HEK 293 cells. (**B**) Correlation measurements between RFP-tagged ß-arrestin 1 with the EGFP-tagged GHSR1a-WT or the mutants were made on a single cell using two coefficients: the PCC and MOC. (**C**) Trafficking of the m-cherry-tagged ß-arrestin 2 with the EGFP-tagged GHSR1a-WT or the mutants GHSR1a DM, GHSR1a -TM or GHSR1a Total expressed in HEK 293 cells. (**D**) PCC and MOC correlation coefficients were evaluated as the measurement of colocalization between m-cherry-tagged ß-arrestin 2 with the EGFP-tagged GHSR1a-WT or the mutants. In (**A**,**C**) confocal images show the localization of the ß-arrestin and the GHSR1a before and following stimulation with ghrelin (100 nM) for 20 or 60 minutes. Receptor fluorescence is shown in green and ß-arrestin fluorescence in red. Co-localisation of ß-arrestin and the receptor is shown in yellow when images are merged. Results are representative of three similar experiments. In (**B**,**C**) The data are expressed as the mean ± SEM.

**Figure 4:**
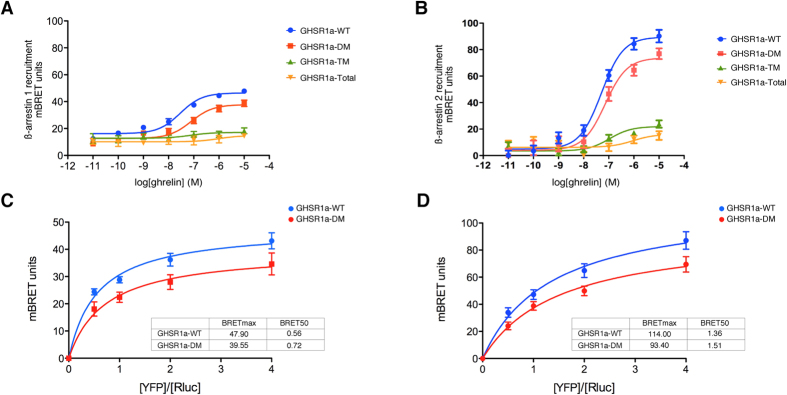
BRET approach to monitor the ß-arrestin interactions with the GHSR1a. GHSR1a-WT and each mutant were used in a BRET based ß-arrestin recruitment assay in HEK 293 cells. Concentration response curves for ghrelin to promote the interaction of GHSR1a with ß-arrestin 1 (**A**) or ß-arrestin 2 (**B**) are shown. (**C**) HEK 293 cells were transfected with a constant amount of ß-arrestin1 and increasing amounts of YFP-GHSR1a-WT or YFP-GHSR1a-DM (acceptors) and treated with ghrelin. Net BRET is expressed as a function of acceptor/donor. (**D**) Titration curves monitoring net BRET in response to varying acceptor/donor ratios were performed as in (**C**). In (**C**,**D**) BRET_50_ and BRETmax values calculated from three independent experiments.

To determine the importance of the receptor C-termini phosphorylation in directing specific signaling events, we first compared ghrelin-induced receptor endocytosis (100 nM) by confocal microscopy in HEK 293 cells expressing GHSR1a-WT or GHSR1a mutants. In the resting cells, fluorescence associated with the receptor was predominantly localised to the plasma membrane (Fig. 2C). A slight fluorescence was also associated with the Golgi apparatus, even after treatment with cycloheximide. After exposure to ghrelin for 20 and 60 minutes, the GHSR1a-WT-associated fluorescence almost completely disappeared from the plasma membrane to become redistributed to a population of intracellular vesicles distributed throughout the cytoplasm (Fig. 2C). In cells expressing GHSR1a-DM, the receptor was primarily distributed throughout the cytoplasm after 20 and 60 minutes of agonist treatment although the population of intracellular vesicles appeared to be reduced (Fig. 2C). By contrast, very little redistribution of the fluorescent labeling could be observed in cells expressing GHSR1a-TM or GHSR1a-Total after 20 minutes and even after 60 minutes of agonist treatment (Fig. 2C). In order to determine whether this change in the patterns of endocytosis displayed by the mutant receptors was due to differences in their ability to interact with ß-arrestins, cells were transiently co-transfected with RFP-tagged ß-arrestin 1 or m-cherry-tagged ß-arrestin 2. As shown in Fig. 3A,B, the receptors (shown in green) were located at the cell surface, whereas RFP-ß-arrestin 1 or m-cherry-ß-arrestin 2 was uniformly distributed in the cytoplasm in unstimulated cells (shown in red). In response to 20-minute stimulation with ghrelin (100 nM), GHSR1a-WT appeared to colocalize with both ß-arrestin 1 and 2 in endocytic vesicles (shown in yellow; Fig. 3A,B). This colocalization is consistent with the assembly of a protein complex containing ß-arrestin and the receptor and appeared to be more robust after 60 minutes of agonist treatment. Similarly, stimulation of GHSR1a-DM induced colocalization of the receptor with both ß-arrestins (Fig. 3A,C). However, this colocalization was rather more evenly distributed in the cytoplasm in a diffuse granular pattern, with no apparent enhancement of localization in endocytic vesicles. In contrast, in the case of GHSR1a-TM and GHSR1a-Total, the receptors remained localized at plasma membrane whilst ß-arrestin remained evenly distributed in the cytoplasm after agonist stimulation (Fig. 3A,C). An examination of two of the coefficients used to quantify the degree of colocalization between fluorophores, the Pearson correlation coefficient (PCC) and the Mander’s overlap coefficient (MOC), supported the assembly of a protein complex containing ß-arrestin and GHSR1a or GHSR1a-DM, and ruled out a complex for GHSR1a-TM or GHSR1a-Total (Fig. 3B,D). To examine in more detail the contributions of agonist dependent phosphorylation in the C-terminal tail of GHSR1a to recruitment of ß-arrestin 1 and 2, BRET assays were performed which enabled association of an eYFP tagged GHSR1a and an Rluc-ß-arrestin to be measured in real time in living cells (Fig. 4A,B, respectively). GHSR1a-WT recruited both ß-arrestin 1 and 2 in an agonist dependent manner. Concentration response curves for ß-arrestin 1 recruitment to each receptor were determined, which revealed that the GHSR1a-DM and GHSR1a-TM showed a subtle but significant decrease in potency compared to GHSR1a-WT (GHSR1a-WT, pEC_50_ = 7.54; GHSR1a-DM, pEC_50_ = 7.10; and, GHSR1a-TM, pEC_50_ = 7.13; p < 0.05). The GHSR1a-Total mutant showed the greatest reduction in potency, which was substantially decreased, compared to GHSR1a-DM (pEC_50_ = 6.01). In addition, there was a significant reduction in efficacy (GHSR1a-DM = 81.3%, GHSR1a-TM = 36.8%, GHSR1a-Total = 31.8% of GHSR1a-WT response; p < 0.05). In the case of ß-arrestin 2, a significant decrease in both potency (GHSR1a-WT, pEC_50_ = 7.30, GHSR1a-DM, pEC_50_ = 7.14; GHSR1a-TM pEC_50_ = 6.97; and, GHSR1a-Total pEC_50_ = 6.04; p < 0.05) and efficacy was also observed (GHSR1a-DM = 82.4%, GHSR1a-TM = 24.6%, GHSR1a-Total = 18.0% of GHSR1a-WT response; p < 0.05). These observations are consistent with the absence of receptor/ß-arrestin colocalization observed by confocal analysis. The BRET values for ß-arrestin 2 recruitment to GHSR1a-WT were greater than those for ß-arrestin 1, which might reflect differences in the receptor/ß-arrestin conformation resulting in a greater distance between the luciferase and YFP tags or difference in affinity of both ß-arrestins.

To gain further insight into the role of receptor phosphorylation in regulating ß-arrestin recruitment, we monitored BRET as a function of the acceptor/donor ratio (eYFP-receptor/luciferase-ß-arrestin) and determined the acceptor-donor ratio at which half-maximal BRET (BRET_50_) is observed (Fig. 3E,F). BRET_50_ values for GHSR1a-DM/ß-arrestin interactions were higher than those for GHSR1a-WT, suggesting that GHSR1a-WT has a higher relative affinity for both ß-arrestins than GHSR1a-DM. The BRET_max_ value was also decreased, indicating that the nature of the receptor/ß-arrestin interactions were different such that the acceptor and donor tags were in closer proximity with the GHSR1a-WT. These data might suggest that the binding of ß-arrestins to the GHSR1a involves two separate sets of interactions with the phosphorylated carboxyl-terminus of the receptor, one with the phosphorylation sites Ser^362^, Ser^363^and Thr^366^ that serve as essential phosphate recognition elements for the ß-arrestin recruitment, and the other with the phosphorylation sites Thr^350^ and Ser^349^ that might stabilize active conformation of ß-arrestins.

### ß-arrestin signaling is determined by the phosphorylation of the C-terminal tail of the GHSR1a: ERK1/2 and Akt activation

**Figure 5:**
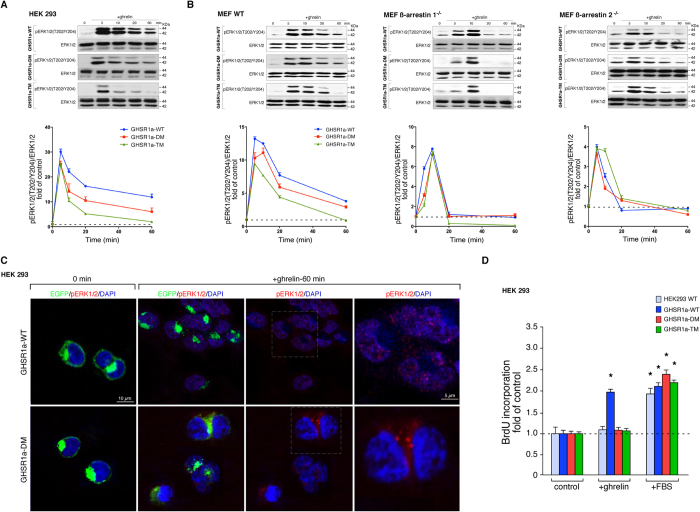
Functional relation between the GHSR1a-associated ß-arrestin-scaffolded complex and the ERK1/2 activity. (**A**) HEK 293 cells were transiently transfected with GHSR1a-WT or mutants and stimulated with ghrelin (100 nM) for the indicated times. Samples of cell lysates were separated by SDS PAGE and immunoblots were performed using anti pERK1/2(T202/Y204) or anti total ERK1/2 antibodies. The levels of pERK1/2were quantified by densitometry, normalized to total ERK1/2 and expressed as the fold change relative to the unstimulated cells. (**B**) The MEF WT, ß-arrestin 1^−/−^ and ß-arrestin 2^−/−^ cells were treated as in (**A**). The levels of pERK1/2(T202/Y204) were expressed as the fold change relative to the unstimulated cells. In (**A**,**B**) immunoblots are representative of three independent experiments, the data are expressed as the mean ± SEM. (**C**) HEK 293 cells expressing GHSR1a-WT and GHSR1a-DM were stimulated with ghrelin for indicated time before being fixed and stained with anti pERK1/2(T202/Y204) antibodies, images were acquired by confocal microscopy. DAPI was used as a counterstain to identify nuclei and is shown in blue. Confocal images are representative of three independent experiments. (**D**) Mitogenic effect of ghrelin (100 nM) on cells transiently transfected with the GHSR1a-WT or mutants (n = 6). Results were expressed as a fold increase in BrDU incorporation relative to control cells. The data are expressed as the mean ± SEM (^*,#^*p* < 0.05).

The role of the two phospho-acceptor regions at the GHSR1a C-terminal tail (Ser^362^, Ser^363^ and Thr^366^, or Thr^350^ and Ser^349^) was first evaluated on ghrelin-induced phosphorylation of ERK1/2 [pERK1/2(T202/Y204)] by transient transfection of GHSR1a-WT, GHSR1a-DM and GHSR1a-TM in HEK 293 cells. In GHSR1a-WT cells stimulated with ghrelin (100 nM), pERK1/2(T202/Y204) was resolved into two components dependent, respectively, on G protein or ß-arrestin signaling as we previously described^11^. G protein-dependent activity was rapid, peaking within ~5 min, followed by a ß-arrestin-dependent activation that was slower in onset, peak ~20 min, and sustained (Fig. 5A). This sustained pERK1/2(T202/Y204) signal declined with C-terminal tail mutations GHSR1a-DM and GHSR1a-TM, whilst the fast and transient G protein-dependent activation was maintained. The decrease in the ERK1/2 activation correlated with the decline of the receptor/ß-arrestin signaling complex formation (Fig. 5A).

These mechanistic differences were confirmed in three types of MEF cells: MEF cells from wild-type mice (MEF WT), MEF cells from ß-arrestin1 null mice (ß-arrestin 1^−/−^) and MEF cells from ß-arrestin 2 null mice (ß-arrestin 2^−/−^) (Fig. 5B). In the MEF WT cells expressing GHSR1a, stimulation with ghrelin led to an early peak phase of ERK activation followed by a sustained plateau phase (Fig. 5B). Whilst the peak ERK activation in response to GHSR1a stimulation in MEF ß-arrestin 1^−/−^ and 2^−/−^ cells was unchanged compared to WT-MEFs, the sustained plateau phase was lost indicating that the plateau phase of the ERK response to GHSR1a was ß-arrestin-dependent. Consistent with this was the observation that expression of the GHSR1a-TM which recruits ß-arrestin poorly resulted in a significant decrease in the plateau phase compared to the wild type GHSR1a and to the mutant GHSR1a-DM, which showed relatively robust coupling to ß-arrestin (Fig. 5B).

We next sought to determine the functional consequences of phosphorylation/ß-arrestin-dependent ERK1/2 activation by the analysis of the mitogenic activity associated with the GHSR1a mutants in HEK 293 cells. Whereas ghrelin-treated GHSR1a-WT cells (100 nM) incorporated BrdU at a ~2-fold over control, cells expressing GHSR1a-DM or GHSR1a-TM failed to incorporate BrdU (Fig. 5D). Whereas the results obtained with the GHSR1a-TM mutant excludes the role of G protein-dependent signaling on ghrelin-activated proliferation, the lack of GHSR1a-DM mitogenic effect might be related to the stabilization of the active conformation of ß-arrestins exerting spatial control over MAPK events. We therefore examined the subcellular location of pERK1/2(T202/Y204) in cells expressing GHSR1a-WT and GHSR1a-DM EGFP-tagged receptors after ghrelin stimulation (100 nM) by confocal microscopy. In cells expressing GHSR1a-DM, pERK1/2(T202/Y204) was primarily observed in the cytoplasm after activation, whereas in cells expressing GHSR1a-WT, most of the pERK1/2(T202/Y204) translocated into the nucleus (Fig. 5C). Thus, the phospho-acceptor sites at the GHSR1a C-terminal tail appeared to influence the ultimate cellular consequence of ß-arrestin recruitment.

**Figure 6:**
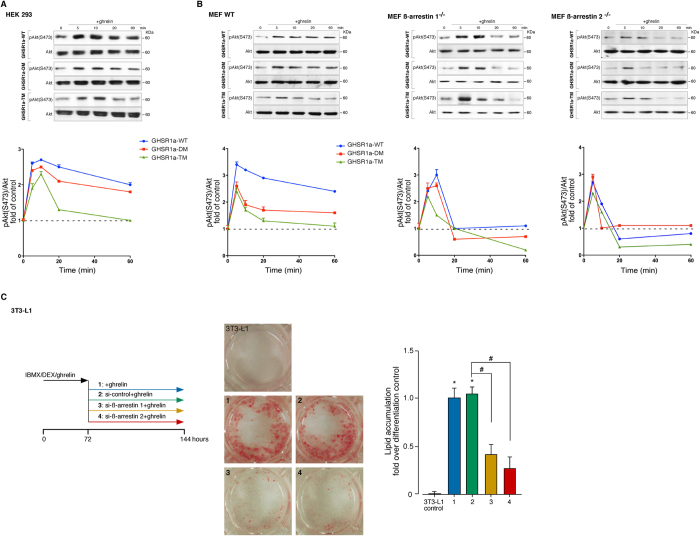
Functional relation between the GHSR1a-associated ß-arrestin-scaffolded complex and the Akt activity. (**A**) HEK 293 cells were transiently transfected with GHSR1a-WT or mutants and stimulated with ghrelin (100 nM) for the indicated times. Samples of cell lysates were separated by SDS PAGE and immunoblots were performed using anti pAkt (S473) or total Akt antibodies. The levels of pAkt (S473) were quantified by densitometry, normalized to total Akt, and expressed as the fold change relative to the unstimulated cells. (**B**) The MEF WT, ß-arrestin 1^−/−^ and ß-arrestin 2^−/−^ cells treated as in (**A**) and the levels of pAkt (S473) were expressed as the fold change relative to the unstimulated cells. In (**A**,**B**) immunoblots are representative of three independent experiments and data are expressed as the mean ± SEM. (**C**) Effect of siRNA depletion of ß-arrestins on terminal adipogenesis in 3T3-L1 cells. After induction for 3 days under treatment with IBMX (0.5 mM), DEX (25 μM), and ghrelin (861 nM) in DMEM/10% FBS, the cells were transfected with specific ß-arrestin 1 or 2 siRNAs and maintained for 3 days in the presence of ghrelin (172 nM) in DMEM/10% FBS. The cells were stained with Oil red O and the lipid droplet accumulation was analyzed using the spectrophotometric absorbance at 520 nm. The results are expressed as the fold change in lipid accumulation relative to the differentiated controls.

The model for the activation of Akt by ghrelin involves the interplay of an early G_i/o_ protein-dependent pathway and a late pathway mediated by ß-arrestins^12^,^13^. Certainly, in HEK 293 cells transiently transfected with the GHSR1a-WT ghrelin (100 nM), ghrelin-activated pAkt (S473) (100 nM) was resolved into two components: an initial rapid G protein-dependent activation which peaks within ∼10 min, this is followed by a ß-arrestin-dependent activation which is sustained over time (Fig. 6A). This sustained pAkt(S473) signal decreased with C-terminal tail mutations GHSR1a-DM and GHSR1a-TM, correlating with the decline of the receptor/ß-arrestin signaling complex formation, this effect was also observed in MEF ß-arrestin 1^−/−^ and 2^−/−^ cells (Fig. 6B). To determine the importance of the GHSR1a C termini in directing specific Akt signaling events, the effects of siRNA-mediated suppression of ß-arrestins were examined on the ghrelin-induced intracellular lipid storage in 3T3-L1 cells. This approach was chosen based on the endogenous GHSR1a expression in either undifferentiated (preadipocytes) or differentiated 3T3-L1 (adipocytes), which would make it difficult to discern the differences among GHSR1a mutants. Thus, 3T3-L1 preadipocyte cells were induced to differentiate into adipocytes using a standard adipogenic induction cocktail of IBMX, DEX and ghrelin for 72 h (early differentiation), followed by suppression of ß-arrestin 1 and 2 with specific siRNAs during terminal differentiation. Oil Red O staining was performed to monitor intracellular ghrelin-induced lipid storage at day 6 after the initiation of differentiation. Efficiency of ß-arrestin 1 and 2 siRNA depletion was confirmed by immunoblot analysis after differentiation (65 ± 5% and 69 ± 2%, respectively). For the ghrelin-induced adipogenesis, depletion of ß-arrestin 1 or 2 caused a substantial inhibition of fat droplet accumulation when compared to siRNA control (61 ± 13% and 73 ± 16%, respectively; Fig. 6C). The ß-arrestin signal complex determines the adipogenic functions of ghrelin highlighting the importance of the phospho-acceptor sites at the GHSR1a C-terminal tail as molecular determinants for the formation of the receptor/ß-arrestin complex and the ultimate signaling outcomes.

### G_q/11_ activity of ghrelin is not related to the phosphorylation of the C-terminal tail of the GHSR1a

**Figure 7:**
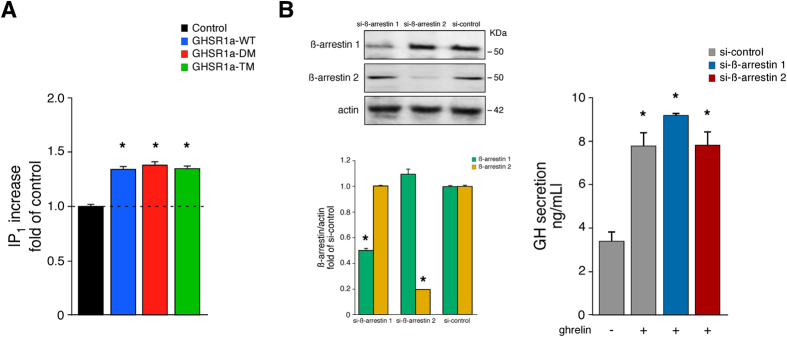
The functionality of G_q/11_-protein mediated GHSR1a signaling is independent of receptor phosphorylation and ß-arrestin binding. (**A**) Determination of ghrelin-induced IP_1_ accumulation in HEK 293 cells transiently transfected with the GHSR1a-WT or mutants. The levels of accumulated IP_1_ were expressed as the fold change relative to the unstimulated GHSR1a-WT cells. Data points correspond to means ± SEM of three experiments performed in triplicate. (**B**) The effect of ghrelin (100 nM) on the GH release in GC cells, transiently transfected with the GHSR1a-WT in the absence or presence of ß-arrestin siRNAs. GH release was estimated by ELISA. Samples of cell lysates were separated by SDS PAGE and immunoblots probed with anti ß-arrestin 1, anti ß-arrestin 2 or anti actin antibodies. The levels of ß-arrestins and actin were quantified by densitometry, normalized to total actin, and expressed as fold relative to the siRNA control. Immunoblots are representative of three independent experiments. The data are expressed as the mean ± SEM (*p < 0.05).

Upon activation, GHSR1a carries information within the cell via a transient increase of intracellular Ca^2+^ through the generation of inositol 1,4,5-triphosphate (IP_3_) triggered by protein subunit G_αq/11_^9^. Because the lifetime of IP_3_ is extremely short, G_αq/11_-dependent GHSR1a activation can be followed by monitoring IP_3_ degradation products, such as inositol 1-phosphate (IP_1_), which accumulates in the cell in the presence of lithium chloride. As shown in Fig. 7A, HEK 293 cells transiently expressing the C-terminal tail mutations, GHSR1a-DM, and GHSR1a-TM, revealed similar IP_1_ accumulation in relation to GHSR1a-WT in response to ghrelin (100 nM). It is well established that the GHSR-1a stimulates GH release through intracellular Ca^2+^ concentration via IP_3_. To further investigate the role of the GHSR1a phosphorylation sites and ß-arrestin signaling, we tested the effect siRNA knockdown of ß-arrestins on the GH release in GC cells. As with 3T3-L1 cells, this approach was selected based on the endogenous GHSR1a expression in GC cells. Transfection of the cells with siRNAs directed against ß-arrestin 1 or 2, which decreased ß-arrestin 1 and 2 expression by 50 ± 1% and 80 ± 3% respectively, did not significantly alter ghrelin-activated GH release compared to cells treated with control siRNA (Fig. 7B). These results demonstrate the contribution of individual G protein and ß-arrestin pathways in the GHSR1a signaling.

## Discussion

In the present study we used mass spectrometry-based proteomic approach to map five phosphorylation sites that can be divided into 2 regions, region 1 (Thr^350^ and Ser^349^) and region 2 (Ser^362^, Ser^363^ and Thr^366^). These regions appear to contribute equally to the overall phosphorylation of the receptor. However we found that the region 2 was primarily responsible for ß-arrestin 1 and 2 binding, receptor internalization, ß-arrestin-mediated ERK and Akt activation. In contrast, region 1 appeared to play a more subtle role of stabilizing the interaction between the receptor and ß-arrestins. In this way our data suggest a differential impact of phosphorylation sites on ß-arrestin recruitment and ß-arrestin-dependent signaling and is consistent with a model in which different phosphorylation pattern (barcode) on the GHSR1a can induce distinct ß-arrestin interactions that determine the ultimate cellular consequences of ß-arrestin signaling.

An intriguing observation was the fact that mutation of the phosphorylation sites Thr^350^ and Ser^349^, GHSR1a-DM, subtly reduced the potency and efficacy of ß-arrestin binding, which might suggest the implication of these phosphorylation elements in the fine-tune of their interactions with the GHSR1a. This modifying role of region 1 on responses such as internalization appeared to be a unique feature of this study. Phosphorylation within region 2 primarily mediated ß-arrestin recruitment and receptor internalization as revealed by the mutant GHSR1a-TM, analysis of ß-arrestin interactions with the region 1 mutant, GHSR1a-DM, determined that ß-arrestin 1 and 2 are both recruited to this mutant receptor but with a lower apparent affinity. These data suggest that although phosphorylation of Ser^362^, Ser^363^ and Thr^366^ can promote ß-arrestin recruitment with the GHSR1a, it is phosphorylation of Thr^350^ and Ser^349^ that is required to stabilize the interaction between the receptor and ß-arrestin. That this stabilization might have functional significance was evidenced by the lack of nuclear translocation of ERK1/2 and cell proliferation observed for the GHSR1a-DM. Importantly, previous studies have determined these GHSR1a functional responses to be dependent on ß-arrestin signaling^11^. Since differences in GPCR-mediated ERK nuclear signaling have previously been linked to the stability of the receptor:ß-arrestin complex^21^,^22^,^23^, it is possible to speculate that the phosphorylation barcode (particularly phosphorylation within region 1) on GHSR1a contributes to the stability of the GHSR1a:ß-arrestin complex in a manner that impacts on ERK nuclear signaling^16^,^24^,^25^.

How might phosphorylation mediate changes in the affinity and stability of the GHSR1a:ß-arrestin complex is not currently clear; however, recent studies using a ß-arrestin biosensor to detect gross changes in conformation suggest phosphorylation of the different sets of sites engenders the distinct functionality of ß-arrestin by inducing different conformations of the receptor-bound ß-arrestin^16^,^24^. These findings are consistent with a model where the patterning of receptor phosphorylation sites establishes a code that determines the conformation of the bound ß-arrestins and subsequently its functional capabilities. Thus, differences in ß-arrestin binding in response to recruitment to GHSR1a *versus* GHSR1a-DM might underlie the differences in signaling we observed.

The interaction between ß-arrestin and activated GPCRs is proposed to involve a biphasic mechanism^26^,^27^,^28^. The first step comprises an interaction between the phosphorylated C-terminal tail of the receptor and the N-terminal domain of arrestin followed by the insertion of the finger loop of ß-arrestin within the receptor core that engages additional binding sites resulting in a longitudinal arrangement on the receptor. Following this model, the phospho-acceptor sites operate in concert with structural elements within the transmembrane core of the receptor. Indeed, recent studies demonstrated that mutations of the contiguous conserved amino acids Pro^148^ and Leu^149^ in the GHSR1a intracellular second loop generate receptors with a strong bias to G protein and ß-arrestin respectively, supporting a role for conformation-dependent signaling bias in the wild-type receptor^29^. Thus, the nature of the active conformation of ß-arrestin and the signaling outcome is determined by the complex ensemble of the GHSR1a phosphorylation sites within the C-terminal tail in combination with structural elements within intracellular loops that confer the functional selectivity.

An interesting aspect is the functional selectivity associated with the differential GHSR1a-stimulated G protein- and ß-arrestin-mediated signaling to control particular cellular response. Our findings from ß-arrestin knockdown indicates the GH-releasing activity results from G protein signaling with no implication of ß-arrestin signaling because this action occurs upon activation of ß-arrestin-impaired GHSR1a. Following the mutation of phospho-acceptor sites, the proliferative effect of ghrelin was impaired implicating ß-arrestin-mediated ERK1/2 pathway in this response. Furthermore, the ß-arrestin-scaffolded complex positively determines Akt activity and adipocyte differentiation. We have previously demonstrated the importance of these scaffolding proteins during ghrelin-induced adipogenesis in 3T3-L1 cells, which determined the expression levels of master regulators of early, the CCAAT/enhancer-binding protein ß (C/EBPß) and the CCAAT/enhancer-binding protein δ (C/EBPδ), and terminal, the peroxisome proliferator-activated receptor (PPARγ) and the CCAAT/enhancer-binding protein α (C/EBPα), adipogenesis^14^. Initially, these findings imply the existence of independent G protein- and ß-arrestin-mediated pathways. However, this does not appear to be the case since ß-arrestin signaling is dependent on the G protein activation^11^,^12^. This fact suggests that certain key components of the G protein-dependent signaling pathways are required to determine ß-arrestin recruitment and signaling. In fact, our previous works demonstrated that ghrelin leads to the activation of Akt through an early G_i/o_-protein-dependent pathway and a late pathway mediated by ß-arrestins^12^,^13^,^14^. The starting point is the G_i/o_-protein dependent PI3K activation that leads to the membrane recruitment of Akt, which becomes tyrosine phosphorylated by c-Src with the subsequent phosphorylation in both the activation loop within the kinase domain [A-loop (T308)] and the hydrophobic motif in the C-terminal region [HM (S473)] by PDK1 and mTORC2, respectively. Once the receptor is activated, a second signaling pathway is mediated by ß-arrestin 1 and 2, involving the recruitment of ß-arrestins, c-Src and Akt. This ß-arrestin-scaffolded complex leads to full activation of Akt. In agreement with these results, assays performed in 3T3-L1 preadipocyte cells indicate that ß-arrestins and c-Src are implicated in the activation of Akt in response to ghrelin through the GHSR1a^12^,^13^. These results support the notion that key components of the G protein-dependent signaling pathways, such as Gi/o-protein dependent Src activation, trigger signaling pathways mediated by ß-arrestins, i.e. full activation of Akt. Additionally, the impact of GRKs or second messenger kinases (i.e. PKCs) on the receptor phosphorylation and consequent ß-arrestin recruitment might be proposed as the missing link between G protein and ß-arrestins. Our data highlight the possibility that the functions of ß-arrestins may be pre-specified by GRK/PKC-receptor interaction. This is consistent with previous works on other GPCRs,, which demonstrate a requirement for GRKs to activate specific transducers as well as to affect transducer functionality in a selective manner^16^,^30^. Thus, the patterning of receptor phosphorylation sites, barcode, could engender subtle differences in ß-arrestin/receptor interactions that lead to divergent ß-arrestin-dependent signaling events.

One of the possible physiological implications of our data is that the signaling outcome of GHSR1a might be determined in a cell type specific manner by differential phosphorylation. For the M3-muscarinic receptor, for example, we have demonstrated that the pattern of receptor phosphorylation varies between different cell types in a manner that might contribute to cell type specific signaling^17^,^18^,^19^,^20^. The fact that we identify two distinct regions of phosphorylation on GHSR1a that contribute differentially to arrestin-dependent signaling means that differential cell type specific phosphorylation might result in different signaling outcomes. We are currently testing this hypothesis by determining if GHSR1a is differentially phosphorylated in different cell types. Ultimately, the fact that GHSR1a can direct functionality via different pathways unveils a tremendous potential for new approaches in developing therapeutics at this receptor particularly taking into account the physiological and pathophysiological effects in both neural and peripheral tissue.

## Experimental Procedures

### Materials

Human ghrelin was obtained from California Peptides (CA, US). Anti-Akt, anti-ERK1/2, anti-pAkt(S473) and anti-pERK1/2(T202/Y204) antibodies were from Cell Signaling Technology (MA, US). Anti-ß-arrestin 1 antibody was obtained from BD Biosciences (CA, US). Anti-EGFP and anti-ß-arrestin 2 antibodies were from Abcam (Cambridge, UK). Secondary antibodies and enhanced chemiluminescence detection system were from GE-Amersham (Buckinghamshire, UK). Radioisotope [^32^P]-orthophosphate (specific activity 8500–9120 Ci/mmol), were from PerkinElmer Life Sciences. Unless otherwise stated, all biochemical and reagents were from Sigma (Mo, US).

### Cell culture

The human embryonic kidney (HEK) 293 wild-type (WT) cell line was maintained in DMEM (Lonza; Basilea, CH) supplemented with fetal bovine serum [FBS, 10% (v/v)], and 100 U/mL penicillin-streptomycin solution (100U/mL). The wild-type (WT), the ß-arrestin 1 knockout (ß-arrestin-1^−/−^) and the ß-arrestin 2 knockout (ß-arrestin-2^−/−^) murine embryonic fibroblast (MEF) cells, provided by Prof. Robert J. Lefkowitz (Duke University Medical Center, Durham, NC, US), were maintained in DMEM supplemented with 10% (v/v) FBS and 100 U/mL penicillin-streptomycin solution. The GC cell line was maintained routinely as a monolayer in complete DMEM medium supplemented with 15% (v/v) horse serum, 2.5% (v/v) FBS and 100 U/mL penicillin G-streptomycin solution. 3T3-L1 preadipocytes were maintained in DMEM containing 10% fetal bovine serum (FBS), 100 U/mL penicillin-streptomycin solution. For 3T3-L1 adipocyte differentiation, the 2-day-postconfluent cells (day 0) were treated with 0.5 mM isobutylmethylxantine (IBMX), 25 μM dexamethasone (DEX), and 861 nM insulin or ghrelin in DMEM containing 10% FBS for 72 h. After 72 h, was renewed every 48 h with DMEM containing 10% FBS and either insulin or ghrelin (172 nM) until the cells were used. Cultures were incubated at 37 °C in a humidified atmosphere containing 5% CO_2_.

### Plasmids, mutagenesis and cell transfection

The wild-type GHSR1a fused at its C terminus to enhanced green fluorescent protein (EGFP) (GHSR1a-WT) in pEFGP-N1 (Clontech, Palo Alto, CA, US) was provided by Prof. Catherine Llorens-Cortes (Institut National de la Sante et de la Recherche Medicale, College de France, Chaire de Medecine Experimentale, Paris, France). EGFP tagged GHSR1a receptor used in the present work showed to have cellular location, endocytosis and G-protein associated signaling similar to the native GHSR1a protein^31^. Mutations to the GHSR1a sequence were incorporated using the QuikChange method (Stratagene, Cheshire, UK), and the identities of all plasmids generated were confirmed through sequencing. The cells were transiently transfected with the GHSR1a-WT and its three mutants [Double mutant (GHSR1a-DM): Thr350Ala, Ser349Ala; Triple mutant (GHSR1a-TM): Ser362Ala, Ser365Ala, Thr366Ala; and, Total mutant (GHSR1a -Total): Thr350Ala, Ser349Ala, Ser362Ala, Ser365Ala, Thr366Ala] using Lipofectamine 2000 (Life Technologies, Invitrogen; Gran Island, NY, US), according to the manufacturer’s instructions. The cell lines expressing the GHSR1a-WT and its three mutants were cultured as above described. Pure cell lines were selected on the basis of resistance to geneticin (G418; 500 μg/mL; maintenance antibiotic). Resistant cells were further selected using flow-assisted cell sorting, after which they were maintained in complete DMEM, supplemented with G418.

ß-arrestin 1 tagged with red fluorescent protein (RFP; ß-arrestin 1-RFP) was provided by Prof. Robert J. Lefkowitz (Duke University Medical Center, Durham, NC, US) through Addgene (Cambridge, MA, US). ß-arrestin 2 tagged with m-cherry or Rluc at its C-terminus (ß-arrestin 2-mcherry) was generated by PROTEX (Protein Expression Laboratory) at the University of Leicester (http://www2.le.ac.uk/departments/biochemistry/facilities/protex). Briefly, ß-arrestin 2 was amplified by PCR using primers, which removed the stop codon and was subcloned into pLeics-30 or pLeics-85 expression vectors. To generate the GHSR1a WT and three mutants fused to enhanced yellow fluorescent protein (eYFP; GHSR1a), receptors were amplified by PCR using primers which removed the stop codon and introduced a 5′ HinDIII and 3′ Kpn I restriction sites. The template for the reaction was GHSR1a WT in pcDNA3.1 vector. The resulting PCR products were subcloned into pcDNA3.1 upstream of full-length eYFP to generate C-terminal eYFP fusion constructs. Mutations were introduced into the C-terminus of the resulting fusion protein using the QuikChange method (Stratagene, Cheshire, UK). Renilla luciferase-tagged ß-arrestin 1 (Rluc-ß-arrestin 1) was provided by Prof. Mark Scott (Institut Cochin, Paris, FR). All cellular assays involving transient transfections were only carried out if the transfection efficiency was 80% or higher.

### [^32^P]Orthophosphate Labelling and GHSR1a Immunoprecipitation

GHSR1a-EGFP, GHSR1a-EGFP DM, GHSR1a-EGFP TM or GHSR1a-EGFP Total cell lines were plated in 6-well plates at 200,000 cells/well 24 h before experimentation and serum starved overnight. For phosphorylation experiments, cells were washed three times with Krebs/HEPES buffer without phosphate [containing in mM: HEPES, 10 (pH 7.4); NaCl, 118; CaCl_2_, 1.3; KCl, 4.3; MgSO_4_, 1.17; NaHCO_3_, 4.17; glucose, 11.7] and incubated in this buffer containing 100 μCi/mL [^32^P]orthophosphate for 1 h at 37 °C and 5% CO_2_. Cells were then stimulated with ghrelin (100 nM, 5 min) and immediately lysed by addition of lysis buffer [containing in mM: Tris/HCl, 20 (pH 7.4); NaCl, 150; and EDTA, 3; and supplemented with 1% (v/v) Nonidet P-40, and 0.5% (w/v) sodium deoxycholate]. GHSR1a was immunoprecipitated from the cleared lysates using GFP-trap (Chromotek; DE) following manufacturer’s instructions. The washed immunoprecipitates were separated by SDS-PAGE on two 10% gels. The first gel was dried, and radioactive bands were revealed using autoradiography film. The second gel was transferred to nitrocellulose membrane as loading control. The analysis was carried out using ImageJ software (National Institutes of Health, Bethesda, MD, US).

### GHSR1a Receptor Purification and Mass Spectrometry

For GHSR1a purification, the GHSR1a-EGFP cell line was harvested (10 confluent T175 flasks), resuspended in Krebs/HEPES buffer and stimulated with ghrelin (100 nM, 5 min). Membranes were then prepared and solubilized by addition of 5 mL of TE buffer plus a mixture of protease and phosphatase inhibitors (Roche Applied Science). After centrifugation at 20,000 × *g*, the resulting supernatant was diluted 1:1 with PBS, and the receptor was then purified on GFP-trap (Chromotek, DE). After extensive washing with solubilization buffer containing 0.5% Nonidet P-40, the resin was resuspended in 2 × SDS-PAGE sample buffer. The sample was resolved by SDS-PAGE on 10% gels and stained with colloidal Coomassie Blue. Purified GHSR1a receptor was excised from the polyacrylamide and washed three times for 5 min with 50 mM ammonium bicarbonate. Reduction and alkylation of cysteines were performed by addition of 10 mM dithiothreitol (DTT) in 50 mM ammonium bicarbonate at 55 °C for 30 min followed by addition 100 mM iodoacetamide in 50 mM ammonium bicarbonate for 30 min in the dark. Gel slices were washed three times for 5 min with 50 mM ammonium bicarbonate containing 50% acetonitrile and incubated overnight at 37 °C in 50 mM ammonium bicarbonate containing 10% (v/v) acetonitrile and 1 μg of sequencing grade trypsin (Promega, Southampton, UK). After tryptic digestion, phosphopeptides were enriched using PHOS-Select^TM^ iron affinity resin.

LC-MS/MS was carried out on each sample using an LTQ OrbiTrap mass spectrometer (Applied Biosystems, Warrington, UK). Peptides resulting from in-gel digestion were loaded at a high flow rate onto a reverse-phase trapping column (0.3 mm inner diameter ×1 mm), containing 5 μm of C18 300 Å Acclaim PepMap media (Dionex, UK) and eluted through a reverse-phase capillary column (75 μm inner diameter ×150 mm) containing Symmetry C18 100 Å media (Waters) that was self-packed using a high pressure packing device (Proxeon Biosystems, Odense, Denmark). The output from the column was sprayed directly into the nanospray ion source of an LTQ Orbital mass spectrometer. The resulting spectra were searched against the UniProtKB/SwissProt data base using MASCOT software (Matrix Science Ltd.) with peptide tolerance set to 5 ppm and the MS/MS tolerance was set to 0.6 Da. Fixed modifications were set as carbamidomethyl cysteine with variable modifications of phosphoserine, phosphothreonine, phosphotyrosine, and oxidized methionine. The enzyme was set to trypsin/proline, and up to two missed cleavages was allowed. Peptides with a Mascot score greater than 20 and where the probability (*p*) that the observed match was a random event was <0.05 were included in the analysis. The spectra of peptides reported as being phosphorylated were interrogated manually to confirm the precise sites of phosphorylation.

### GHSR1a/ß-arrestin Interaction Assays

A bioluminescence resonance energy transfer (BRET) assay was used to monitor interactions between GHSR1a and ß-arrestin. YFP tagged-GHSR1a-WT (YFP-GHSR1a-WT), YFP-GHSR1a-DM, YFP-GHSR1a-TM or YFP-GHSR1a-Total were co-transfected with Rluc-ß-arrestin 1 or Rluc-ß-arrestin 2 at a ratio of 4:1 using Lipofectamine 2000 (Life Technologies, Invitrogen, Gran Island, NY, US) following manufacturer’s indications. After 24 h incubation, cells were subcultured into poly-D-lysine-coated white 96-well microplates, incubated for a further 24 h prior to the assay and serum starved overnight. Cells were then washed with Hanks’ balanced salt solution and incubated in this buffer for 30 min prior to conducting the assay. To initiate the assay, the Rluc substrate coelenterazine (Life Technologies, Invitrogen; Gran Island, NY, US) was added to a final concentration of 2.5 μM and incubated for 10 min at 37 °C before ghrelin was added. Following a further 5 min incubation, luminescence emissions at 535 and 475 nm were measured using a CLARIOstar (BMG Labtech; Offenburg, DE), and the BRET signal was presented as the 535/475 ratio multiplied by 1000 to yield the arbitrary milli-BRET units.

### Immunoblot analysis

Serum-starved cells were stimulated with ghrelin (100 nM) for the indicated time period at 37 °C. The medium was then aspirated and the cells were lysed in ice-cold RIPA buffer supplemented with protein and phosphatase inhibitors. The solubilized lysates were transferred into centrifuge tubes and left at 4 °C for 15 min, then pre-cleared by centrifugation at 18,000 × *g* for 15 min at 4 °C. Protein concentration was evaluated with the QuantiPro BCA assay kit. Subsamples (same amount of protein) of each sample were separated by SDS-PAGE on 10% gels and transferred to nitrocellulose membranes. The immunoreactive bands were detected by enhanced chemiluminescence (Pierce ECL Western Blotting Substrate; Thermo Fisher Scientific, Pierce, Rockford, IL, US). The resulting protein bands were scanned and analyzed using ImageJ software (National Institutes of Health, Bethesda, MD, US) and normalized for the corresponding loading controls.

### Confocal Assays

For analysis of the endocytosis time course, HEK 293 cells on poly-D-lysine-coated coverslips were transfected with the EGFP-tagged GHSR1a-WT, GHSR1a-DM, GHSR1a-TM or GHSR1a-Total. The cells were grown overnight in a humidified atmosphere of 95% air and 5% CO_2_ at 37 °C. The cells were preincubated for 120 min at 37 °C with 90 μM cycloheximide in all experiments to prevent *de novo* protein synthesis. The cells were preincubated for 30 min at 4 °C in ice-cold Earle’s buffer [containing (in mM): 140 NaCl, 5 KCl, 1.8 CaCl_2_, and 3.6 MgCl_2_ (pH 7.4); and, complemented with 0.2% BSA, 0.01% glucose, 90 μM cycloheximide, and 0.8 mm of 1–10 phenanthrolene] in the presence/absence of ghrelin (100 nM). Internalization was promoted by placing the cells at 37 °C. The cells were then rinsed three times with ice-cold Earle’s buffer and subsequently fixed for 10 min with 4% paraformaldehyde dissolved in 0.1 mM phosphate-buffered saline [PBS (pH 7.4)]. The cells were rinsed again in cold Earle’s buffer, mounted using Vectashield (Vector Laboratories, Compiègne, France). To determine the interaction between the GHSR1a and ß-arrestins, the HEK 293 cells were cotransfected with the EGFP-tagged GHSR1a-WT, GHSR1a-DM, GHSR1a-TM or GHSR1a-Total and the corresponding RFP-tagged ß-arrestin 1 or m-cherry-tagged ß-arrestin 2 in a 1:1 ratio. For immunofluorescence analysis of ERK1/2 activation, the HEK 293 cells were transfected and cultured as indicated above. After ghrelin stimulation for the indicated times, the cells were fixed with 4% buffered paraformaldehyde-PBS for 15 min, washed, permeabilized (1% Triton X-100, 1% Tween 20 in PBS) for 30 min, and blocked with PBST (1% Triton X-100, 1% Tween 20, 5% heat-inactivated normal goat serum, 0.2% BSA in PBS) for 60 min and then incubated with anti-pERK1/2(T202/Y204) antibody diluted in PBST (1:500) overnight at 4 °C. After three washes with PBS, cells were incubated with the secondary antibody (Alexa Fluor 594-conjugate goat anti-rabbit antibody) in PBST (1:1000) for 60 min at room temperature. DAPI was used to counterstain the cell nuclei (Invitrogen). Digital images of cell cultures were acquired with a Leica TCS-SP5 spectral confocal microscope (Leica Microsystems; Heidelberg, DE). Quantification of PCC and MOC was developed using ImageJ software (National Institutes of Health, Bethesda, MD, US).

### Proliferation assays

HEK 293 cell proliferation was determined using the BrdU incorporation-ELISA assay (Roche Applied Science; Mannheim, GE) following manufacturer’s instructions. Briefly, cells (10 × 10^3^/well) were cultured in DMEM supplemented with ghrelin (100 nM) for 12 h and then were incubated with 10 mg/mL BrdU for 12 h before being fixed with FixDenat solution. The fixed cells were further treated with anti-BrdU-POD working solution, and rinsed with washing solution before substrate solution was added. The absorbance at 370 nm (reference wavelength at 492 nm) was measured using an ELISA plate reader (Reader VersaMaxPLUS).

### Quantification of lipid accumulation

The accumulated lipid droplets were stained with Oil Red O as previously described^14^. Briefly, the differentiated 3T3-L1 adipocytes cells were washed with PBS, fixed with formaldehyde solution and stained with Oil Red. For quantification, the cells were washed extensively with water to remove unbound dye, isopropanol was added to the stained culture plates, and analyzed using spectrophotometry at 520 nm.

### Inositol 1-phosphate incorporation

Inositol 1-phosphate (IP_1_) incorporation was measured using IP-One HTRF^®^ assay kit (Cisbio Assays, MA, USA) based on FRET technology. GHSR1a-WT, GHSR1a-DM, GHSR1a-TM or GHSR1a-Total cell lines were cultured into poly-D-lysine-coated 96-well microplates, incubated for a further 24 h prior to the assay and serum starved overnight. Cells were then treated with stimulation buffer plus ghrelin (100 nM, 1 h at 37 °C) and lysed by addition of lysis buffer for 30 min with shaking at 500 rpm. To initiate the assay 16 μL of lysate was transferred to a white 384 well plate and incubated with IP1-d2 conjugate and Anti-IP1 cryptate Tb-conjugate (1 h at 500 rpm). Fluorescence emissions at 665 and 620 nm were measured using a CLARIOstar (BMG Labtech; Offenburg, DE).

### GH assay

Murine GH was measured by a rat/mouse GH ELISA (EMD Millipore, Billerica, Massachusetts, USA) in medium collected from the GC cultures, post-transfected with siRNAs according to the manufacture’s instructions. GH levels were analyzed in quadruplicate.

### Small interfering RNA (siRNA) silencing of gene expression and transfection

Chemically synthesized double-stranded siRNA duplexes for ß-arrestin 1 and ß-arrestin 2 were selected from ON-TARGET plus SMART pool siRNA from Thermo Fisher Scientific (Dharmacon, Lafayette, CO, US; mouse ß-arrestin 1 (5′ → 3′): ACGGGAAGCUCAAGCAUGA; UCAUAGAGCUUGACACCAA; GGAGAACCCAUCAGCGUUA; UGGAUAAGGAGAUCUAUUA; human ß-arrestin 1 (5′ → 3′): UGGAUAAGGAGAUCUAUUA, AUGGAAAGCUCACCGUCUA,GAACUGCCCUUCACCCUAA,GAACGAGACGCCAGUAGAU; mouse ß-arrestin 2 (5′ → 3′): GUGCCAAAACAAUAGAAGA; AUACCAACCUCAUCGAAUU;CUACUUGAAGGACCGGAAA;GGGCCUGUCUUUCCGCAAA; human ß-arrestin 2(5′→3′): CGAACAAGAUGACCAGGUA,CGGCGUAGACUUUGAGAUU,GGGCUUGUCCUUCCGCAAA,UAGAUCACCUGGACAAAGU. An ON-TARGET plus Non-targeting siRNA was used as a control for all siRNA experiments. The cells were transfected with Lipofectamine 2000 (Life Technologies, Invitrogen, Gran Island, NY, US) according to the manufacture’s instructions. Based on the short half-life of these siRNAs during adipocyte differentiation (~4 days), the confluent 3T3-L1 cells were transfected after the induction of adipogenesis [transfection in terminal adipocyte differentiation: treatment with 0.5 mM IBMX, 25 μM DEX, and 861 nM ghrelin in DMEM containing 10% FBS for 3 days for induction of adipogenesis, and then siRNA transfection and maintenance in DMEM containing 10% FBS supplemented with 172 nM ghrelin for 3 days as previously described^14^.

### Data analysis

All values are presented as mean ± standard error of the mean (SEM). Student *t* test were performed to assess the statistical significance of 2-way analysis. For multiple comparisons, ANOVA was employed. *p* < 0.05 was considered as statistically significant (^*,#^).

## Additional Information

**How to cite this article**: Bouzo-Lorenzo, M. *et al.* Distinct phosphorylation sites on the ghrelin receptor, GHSR1a, establish a code that determines the functions of ß-arrestins. *Sci. Rep.* **6**, 22495; doi: 10.1038/srep22495 (2016).
